# A novel method for measuring diet-induced thermogenesis in mice

**DOI:** 10.1016/j.mex.2019.08.016

**Published:** 2019-09-10

**Authors:** Tomomi Yamazaki, Reina Ikaga, Dongyang Li, Satoshi Nakae, Shigeho Tanaka

**Affiliations:** a Department of Clinical Nutrition, National Institute of Health and Nutrition, National Institutes of Biomedical Innovation, Health and Nutrition, 1-23-1 Toyama, Shinjuku-ku, Tokyo, 162-8636, Japan; b The Graduate School of Humanities and Sciences, Ochanomizu University, 2-1-1 Otsuka, Bunkyo-ku, Tokyo, 112-8610, Japan; c Graduate School of Engineering Science, Osaka University, 1-3 Machikaneyama, Toyonaka, Osaka, 560-8531, Japan; d Department of Nutrition and Metabolism, National Institute of Health and Nutrition, National Institutes of Biomedical Innovation, Health and Nutrition, 1-23-1 Toyama, Shinjuku-ku, Tokyo, 162-8636, Japan

**Keywords:** Measurement of diet-induced thermogenesis in mice, Energy expenditure, Mice, Physical activity, Thermic effect of food

## Abstract

Diet-induced thermogenesis (DIT) refers to energy expenditure (EE) related to food consumption. Enhancing DIT can lead to weight loss. Factors that increase DIT are expected to lower body mass index and body fat mass. Although various methods have been developed for measuring DIT in humans, there is currently no method available for calculating absolute DIT values in mice. Therefore, we attempted to measure DIT in mice by applying the method more commonly used for humans. Mouse energy metabolism was first measured under fasting conditions; EE was plotted against the square root of the activity count, and a linear regression equation was fit to the data. Then, energy metabolism was measured in mice that were allowed to feed ad libitum, and EE was plotted in the same way. We calculated the DIT by subtracting the predicted EE value from the fed EE value for the same activity count. The methodology for measuring DIT in mice may be helpful for researching ways of combatting obesity by increasing DIT.

• The methodology for measuring absolute DIT values in mice is developed.
• For mice, the proportion of DIT compared with calorie intake and EE are 12.3% and 21.7%, respectively.

## Graphical abstract

**Figure:**
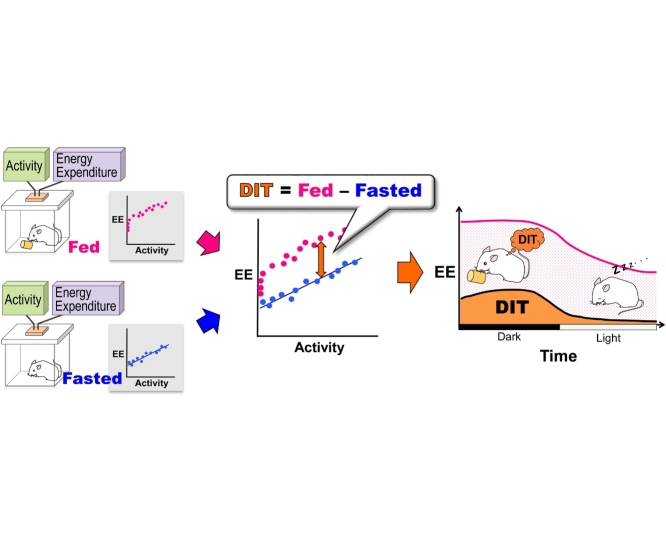

**Specifications Table**

**Table:** 

Subject Area:	Agricultural and Biological Sciences
More specific subject area:	Energy metabolism
Method name:	Measurement of diet-induced thermogenesis in mice
Name and reference of original method:	NA
Resource availability:	NA

## Method details

### Animals

Seven-week-old male ddY mice (n = 6) were obtained from Japan SLC, Inc. (Hamamatsu, Japan) and fed a normal laboratory diet (CE2; Clea, Tokyo, Japan) for at least 1 week to stabilize their metabolic condition until use. Mice were exposed to a 12-h light (from 0700 to 1900 h)/12-h dark (from 1900-0700 h) cycle, and the room was maintained at a constant temperature of 22 °C. They were individually housed and allowed free access to experimental diets and water. Mice were cared for in accordance with the National Institutes of Health's Guide for the Care and Use of Laboratory Animals. All animal procedures were reviewed and approved by the National Institutes of Biomedical Innovation, Health and Nutrition, Japan (No. DS27-52).

### Statistical analysis

Values are shown as means ± SEM. When a Levene test showed that the data met the assumption of homogeneity of variances, we performed a Tukey HSD *post hoc* test (IBM SPSS Statistics 23). When the data did not meet the assumption of homogeneity of variances, we performed a Games-Howell *post hoc* test. Statistical significance was set at *P* <  0.05.

### Method for calculating diet-induced thermogenesis

The diet was prepared as described in our previous study [1]. The composition of the diet, expressed as the percentage of total calories, was 10% fat, 20% protein, and 70% carbohydrate. The mice consumed 21.6 ± 0.6 kcal per day *ad libitum*.

Open-circuit indirect calorimetry was performed with an O_2_/CO_2_ metabolism measuring system for small animals (MK-5000RQ; Muromachi Kikai Co., Ltd., Tokyo, Japan). The system monitored VO_2_ and carbon dioxide production (VCO_2_) at 3-min intervals. Spontaneous motor activity, expressed as the number of times that the animal crossed the infrared rays per minute, was measured at 3-min intervals using a Supermex infrared sensor (Muromachi Kikai Co., Ltd.).

Mice entered the calorimeter without food at 1700 h, and energy metabolism was measured for 11 h (from 0000 to 1100 h). The energy expenditure (EE) rate was calculated using the formula described by Ferrannini, in which the rate of EE (kcal/min) = 3.91 VO_2_ + 1.10 VCO_2_ − 3.34 N, where N is the rate of urinary nitrogen excretion used to estimate protein oxidation [2]. However, given that only a small portion of the resting and exercise energy expenditure arises from protein oxidation, the contribution of protein oxidation was disregarded, and the following equation was used to calculate EE: EE (kcal/min) = 3.9 VO_2_ + 1.1 VCO_2_ [3].

For the fed measurements, the same mice used for the fasted measurements entered the calorimeter at 1600 h, the research diets were provided at 1700 h, and energy metabolism was measured for 22 h (from 1700-1500 h).

The system monitored VO_2_, VCO_2_, and activity at 3-min intervals; every four data points were averaged, and the mean value was used as the average value for the 12-min period. The data were normalized to the square root of the activity count. Fifty-five (5/h x11 h) values each for EE and activity were selected from the measurements taken over 11 h under fasting conditions, we plotted EE against the square root of activity and identified a linear regression equation by simple linear regression analysis. Then, 110 (5/h x22 h) values each for EE and activity were selected from the measurements taken over 22 h under fed conditions and we plotted EE against the square root of activity.

**Fig. 1:**
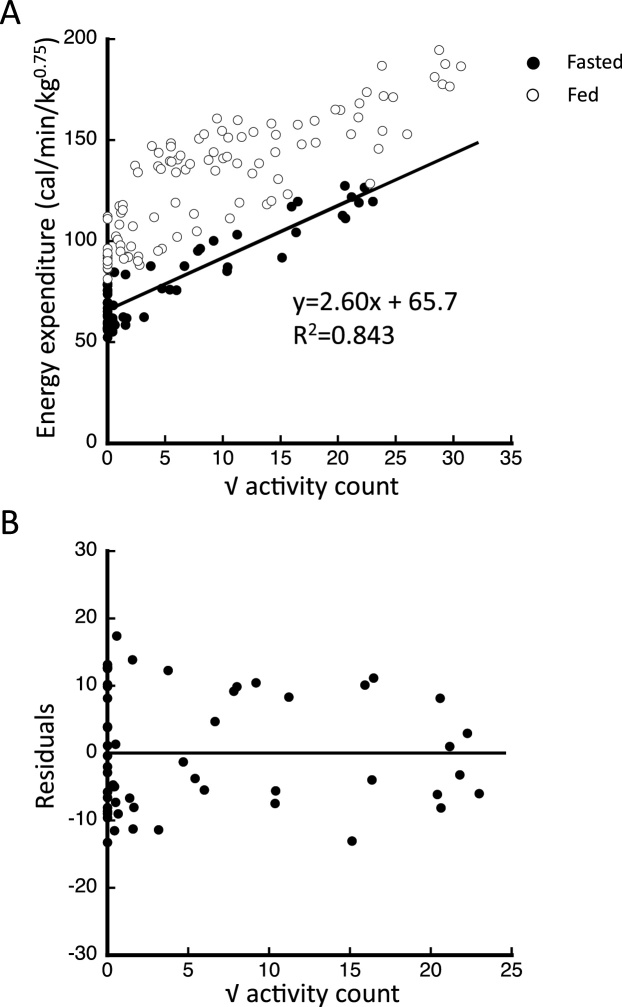
Scatter plots of the square root of activity count *vs*. energy production under fasted and fed conditions, obtained from one representative ddY mouse. The scatter plots acquired under fasted and fed conditions and the regression line found for the fasted data points are shown in (A), and the residual plot is shown in (B). Closed and open circles indicate fasting and feeding data points, respectively.

Fig. 1A shows representative scatter plots of the square root of the activity count and EE under two conditions—fasting and fed—using data acquired from a single mouse. The regression equation and the R^2^ value calculated from the fasting data are also illustrated in Fig. 1A. The R^2^ value ranged from 0.650 to 0.843 for the six mice that were assessed. When scatter plots were generated using the activity count (not the square root of activity count) and EE, R^2^ value ranged from 0.600 to 0.808. Because the scatter plots fitted better when the x-axis represented the square root of that activity count rather than the activity count, we adopted the former approach. Residual plots of the fasting data shown in Fig. 1A are shown in Fig. 1B. The plot showed homoscedasticity, indicating that our data set was most likely a good fit for regression. For the mice that do not move much, plotting the logarithmic transformation of each activity count on the x-axis resulted in a better fit because of the most of the data points clustered on the smaller values of activity counts.

**Fig. 2:**
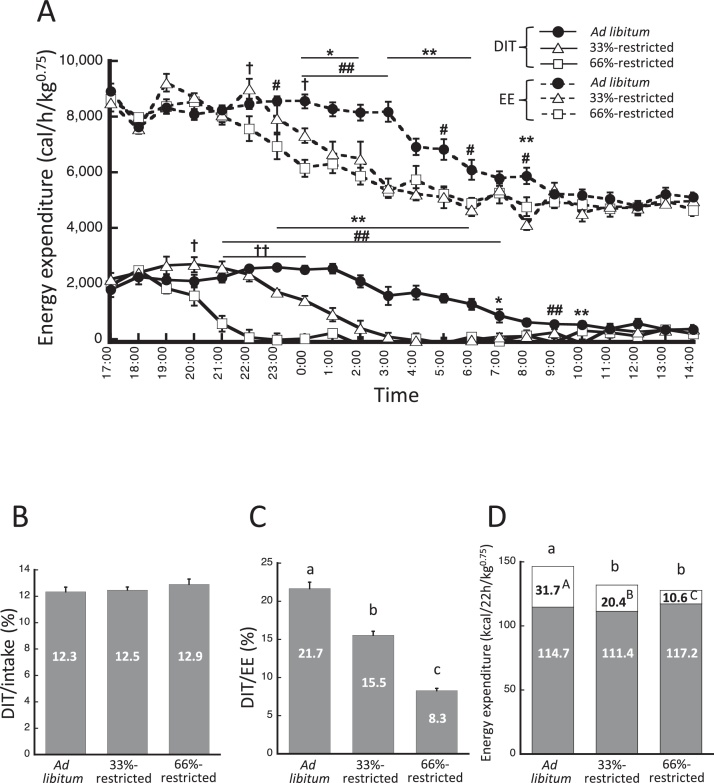
The DIT calculated for mice. Time course of DIT and energy expenditure (A). Straight and dotted lines indicate DIT and energy expenditure, respectively. Closed circles, open triangles, and open squares indicate data from the *ad libitum* group, and the 33%- and 66%-restricted groups, respectively. DIT_/intake_ (B), DIT_/EE_ (C), and total energy expenditure (D). The **white** portions of the columns indicate DIT. The gray portions of the columns indicate the values obtained by subtracting DIT from total energy expenditure. Values are shown as the mean ± SEM (n = 6). *P < 0.05 and **P < 0.01; *ad libitum vs*. 33%-restricted. ^#^*P* <  0.05 and ^##^*P* < 0.01; *ad libitum vs*. 66%-restricted. ^†^*P* < 0.05 and ^††^*P* < 0.01; 33%-restricted *vs*. 66%-restricted. Means without a common letter are significantly different (*P* <  0.05).

The difference between the fed EE value and the predicted EE value for the same activity count represents the diet-induced thermogenesis (DIT). Fig. 2A shows the change in DIT and EE in the *ad libitum* group and the groups restricted by 33% or 66% calories (33%-restricted and 66%-restricted, respectively) over 1-h intervals for 22 h. After the measurements under fasting conditions, mice were fed *ad libitum* for one week and we measured energy metabolism under 66%-restricted, *ad libitum*, and 33%-restricted conditions, on the first, the second, and the third day, respectively. For the calorie restriction study, mice were given only 15 kcal of the indicated diet per day (33%-restricted group) or 7.5 kcal of the indicated diet per day (66%-restricted group). The DIT remained high after the mice were allowed to eat and then gradually decreased. The DIT for the 66%-restricted mice began to decrease 3 h after they started to eat because all of the food had been consumed by this time. The DIT of the 33%-restricted mice began to decrease 6 h after they started eating. The *ad libitum* mice maintained a high DIT for 10 h after they started eating. The EE decreased earliest in the 66%-restricted group, followed by the 33%-restricted group, and then the *ad libitum* group. The total activity counts during the dark cycle were 180.6 ± 16.3, 190.3 ± 27.6, and 296.6 ± 39.9 counts/min for the *ad libitum*, 33%-, and 66%-restricted mice, respectively (*P* = 0.035, for *ad libitum vs*. 66%-restricted). The total activity counts during the light cycle were 63.8 ± 5.1, 56.9 ± 12.1, and 75.1 ± 7.5 counts/min for the *ad libitum*, 33%-, and 66%-restricted mice, respectively, and there were no significant differences. The total DIT and EE over 22 h were calculated from the area under each curve. DIT (%) *versus* calorie intake was calculated by dividing the total DIT by the total calorie intake, and is shown as DIT_/intake_ in Fig. 2B. The DIT_/intake_ values were 12–13% for all three groups, and there are no significant differences. DIT (%) *versus* EE was calculated by dividing the total DIT by the total EE, and is shown as DIT_/EE_ in Fig. 2C. The DIT_/EE_ value for the *ad libitum* mice, 21.7%, was significantly higher than those for the other two groups. The DIT_/EE_ for the mice fed the 66%-restricted diet was significantly lower than that of the others. The mice fed the 33%- and 66%-restricted diets exhibited DIT values that were 28.6% and 61.7% less, respectively, than that of the *ad libitum* mice. Fig. 2D shows the DIT and total daily energy expenditure (TEE) values for all three groups. The *ad libitum* mice had the highest TEE out of the three groups because they had the highest DIT.

### Method validation

From the diet-restricted study, we found that, in mice, the lower the energy intake, the lower the DIT. A mixed model meta-regression analysis of 19 studies of human DIT showed that a higher energy intake increased DIT, and that for every 100 kJ increase in energy intake, the DIT increased by 1.1 kJ/h [4]. The value of DIT_/intake_ for mice was close to that reported for humans, which is 10% [5]. In humans, the main determinants of DIT are the energy content and the protein fraction of the diet [5]. Although we did not examine the DIT produced by each nutrient fraction of the diet, it has been previously reported that protein, carbohydrate, and fat intakes have similar thermogenic effects in mice [6]. The value of DIT_/EE_ identified for mice in this study was more than 20%, which is twice as high as that reported for humans (10% of TEE) [5,[7], [8], [9]]. Over the course of our study, the mice gained 3–5% of their body weight (BW) per week. Based on the results shown in Fig. 2C, if mice were fed a 50%-restricted feeding, they would most likely exhibit approximately 12% DIT_/EE_. Thus, in these mice, overeating appears to have contributed to weight gain. The EE per BW in mice is reported to be seven times higher than that in humans, which is attributed to the higher mitochondrial density [10]. This means that DIT_/EE_ per BW in mice is fourteen times higher than that in humans. In this study, we used mice with a BW of 38.8 ± 1.1 g that consumed 21.6 ± 0.6 kcal/day *ad libitum* (0.557 kcal/g BW). Based on the Dietary Reference Intakes for Japanese (2015), the BW and estimated energy requirement for 18-29-year-old Japanese males are 63 kg and 2650 kcal/day, respectively (0.0421 kcal/g BW) [11]. For adults with balanced energy intake and output, the estimated energy requirement must be equal to energy intake. Since the DIT_/intake_ is about 10% for both humans and mice, the DIT_/EE_ per BW in mice is about thirteen times higher than that in humans. This value is almost identical to that calculated above.

**Fig. 3:**
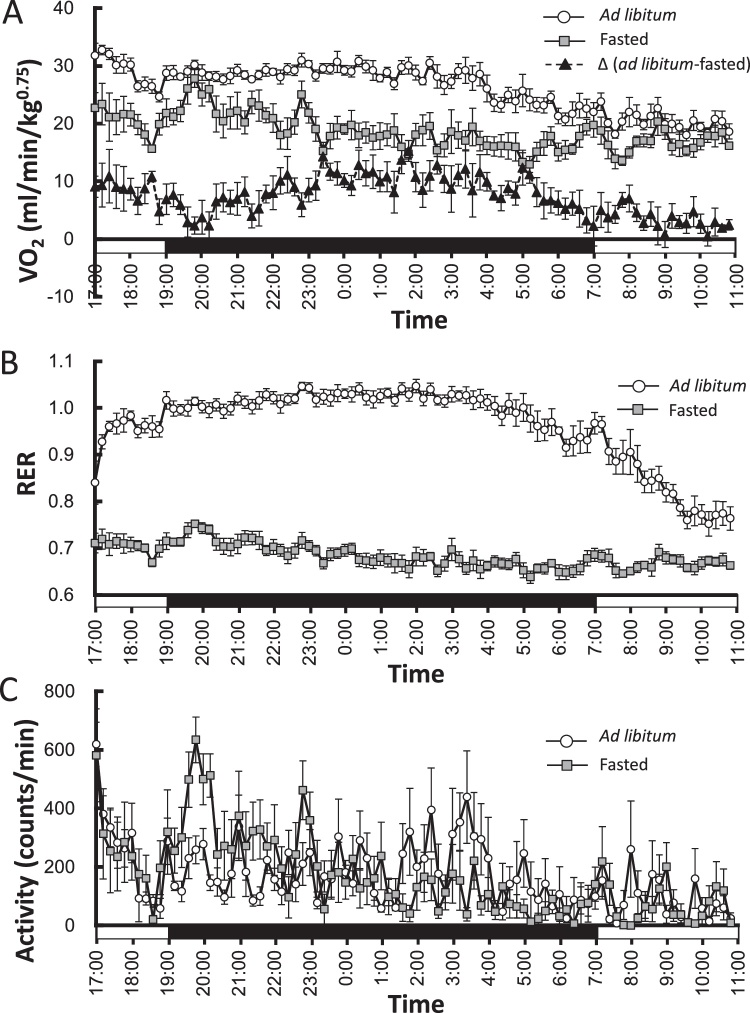
Oxygen consumption, respiratory exchange ratio, and activity counts. The oxygen consumption of mice that were allowed to feed *ad libitum* (open circle) or were fasted (gray square), and the difference between these two values (closed triangle) (A). Respiratory exchange ratio (RER) of mice that were allowed to feed *ad libitum* (open circle) or were fasted (gray square) (B). Activity counts for mice that were allowed to feed *ad libitum* (open circle) or were fasted (gray square) (C). The black and white bars indicate dark and light cycles, respectively. Values are shown as the mean ± SEM (n = 6).

To estimate increases in DIT, body temperature and VO_2_ are traditionally observed [6,[12], [13], [14]]. The results from measuring the increase in VO_2_ are shown in Fig. 3A. Respiratory exchange ratio (RER) is also shown in Fig. 3B. When mice were allowed to feed *ad libitum*, the VO_2_ and RER increased as soon as they started eating. However, 1.5 h from when they started feeding, the VO_2_ decreased as their activity decreased (Fig. 3A and C). The difference between the fed (*ad libitum*) and fasting VO_2_ values decreased further after 2.5 h from when the mice started feeding, because the fasted mice exhibited increased activity levels during the time that they expected to be fed. The RER values of fasted mice are also increased at 1930 h. Thus, increases in VO_2_ are dependent not only on feeding but also on mouse activity levels and it is very important to consider activity when calculating increases in EE or DIT, as shown here.

## Additional information

### Background information

DIT, also known as the thermic effect of food, is defined as the increase in EE above the fasting state and is related to processes including digestion, intestinal absorption of nutrients, and storage of the absorbed nutrients [7]. The TEE includes the basal metabolic rate (BMR), DIT, and physical activity-related energy expenditure [8]. DIT values are commonly expressed as a percentage of the energy content of the food ingested. Typical DIT values are reported to be 5%–10% for carbohydrates, 0%–3% for fats, and 20%–30% for proteins [5,7,8]. In healthy subjects with a mixed diet, DIT over a 24-h period represents about 10% of the total amount of energy ingested [5]. When a subject is in energy balance, where intake equals expenditure, DIT is 10% of TEE [5,[7], [8], [9]]. DIT in humans is generally measured using ventilated hood systems [[15], [16], [17]] or respiratory chambers [9,[18], [19], [20]]. Using a respiratory chamber to measure DIT has the advantage of simulating real-life conditions over an entire day. Schutz et al. first calculated DIT by subtracting BMR from the intercept of the linear regression between EE *versus* physical activity (PA) frequency (*i.e.*, energy expenditure at zero activity) during the postprandial state, as assessed using a radar system in a respiratory chamber [18]. However, Tataranni et al. indicated that measuring DIT using Schutz’s method is not ideal due to poor reproducibility arising from the large variability in the terms used to calculate DIT (that is, BMR and the intercept of the regression line between EE and PA) [19]. Moreover, they pointed out that the method proposed by Schutz et al. underestimated DIT [19]. Hence, Tataranni et al. proposed calculating the DIT as the difference in EE between the fed state and the fasted state [19]. Although this method requires taking two separate measurements, some consider this method the gold standard for estimating the DIT [21].

In mice, DIT has been estimated by measuring the postprandial increase in body temperature, *e.g.*, rectal or dorsal temperature, and the increase in oxygen consumption (VO_2_) [6,[12], [13], [14]]. These methods enable us to compare the magnitude of DIT induced in mice fed a variety of experimental diets or in mutant mice. However, absolute DIT values cannot be calculated using these methods. Moreover, the ratio of DIT to energy intake or to TEE is rarely calculated. Because enhancing DIT may lead to weight loss [22], various foods and their ingredients have been widely studied for their ability to enhance DIT in humans. To date, capsaicin, catechins, and caffeine have been reported to increase DIT [[23], [24], [25]]. Developing a method for measuring DIT in mice would make it possible to research foods that increase DIT and protect against obesity and elucidate the underlying mechanism. In this study, we developed a novel technique for measuring absolute DIT values in mice by applying methodologies use to measure DIT in humans to mice.
